# Three-month early change in prostate-specific antigen levels as a predictive marker for overall survival during hormonal therapy for metastatic hormone-sensitive prostate cancer

**DOI:** 10.1186/s13104-021-05641-5

**Published:** 2021-06-03

**Authors:** Shotaro Nakanishi, Masato Goya, Mitsuyoshi Tamaki, Takuma Oshiro, Seiichi Saito

**Affiliations:** 1 Department of Urology, Graduate School of Medicine, University of the Ryukyus, 207 Uehara, Nishihara, Okinawa 903-0215 Japan; 2 Naha City Hospital, Naha, Okinawa Japan

**Keywords:** mHSPC, Time to CRPC, Prostate cancer, 3-Month %PSA

## Abstract

**Objective:**

To date, there are no useful markers for predicting the prognosis of metastatic hormone-sensitive prostate cancer (mHSPC). We evaluated the effect of early changes in prostate-specific antigen (PSA) levels after androgen deprivation therapy (ADT) on castration-resistant prostate cancer (CRPC) progression and overall survival (OS) in mHSPC patients.

**Results:**

In 71 primary mHSPC patients treated with ADT, the median times to CRPC and OS were 15 months and 92 months, respectively. In multivariate analysis, a Gleason score of ≥ 8 (p = 0.004), an extent of disease value (EOD) of ≥ 2 (p = 0.004), and a 3-month PSA level > 1% of the pretreatment level (p = 0.017) were independent predictors of shorter time to CRPC. The area under the receiver operating characteristic curve was feasible at 0.822. A 3-month PSA level > 1% of the pretreatment level was an independent predictor of OS (p = 0.004). Three factors were independent predictors of shorter time to CRPC. A 3-month PSA level > 1% of the pretreatment level correlated with a poor prognosis.

**Supplementary Information:**

The online version contains supplementary material available at 10.1186/s13104-021-05641-5.

## Introduction

Androgen deprivation therapy (ADT) has been the standard of care for metastatic hormone-sensitivity prostate cancer (mHSPC). However, the effects of ADT on mHSPC vary substantially; some patients show early resistance while the others show long-term effects [1, 2]. Consequently, research into the mechanism of early resistance includes both clinical analyses, such as for metastatic burden, and molecular analyses, such as on the androgen receptor [3–5]. To date, there are no useful markers for predicting the prognosis of prostate cancer in clinical practice or trials.

Docetaxel and abiraterone acetate were shown to increase the survival rate of men commencing ADT for mHSPC [6–9]. As a result, abiraterone acetate has been administered in Japan since 2018 for high-risk cases of mHSPC (those that satisfy two or more of a Gleason score ≥ 8, visceral metastasis, and ≥ 3 bone metastases). However, problems or adverse events associated with long-term use of docetaxel or abiraterone acetate for mHSPC have not been resolved. In addition, some prostate cancer patients, particular Asian patients, who receive chemotherapy, experience severe toxicities due to drug tolerability [10, 11]. There is no established index for predicting the prognostic factors for high-risk patients.

Several biomarker candidates associated with the prognoses of ADT-treated mHSPC patients have been identified [12], and risk stratification models for mHSPC using these biomarkers have been proposed [4, 12]. Studies show that prostate-specific antigen (PSA) half-life and doubling time have a greater correlation with clinical outcomes in patients with mHSPC and/or castration-resistant prostate cancer (CRPC) than that of pretreatment variables [13]. Additionally, there have been reports about the relationship between the velocity of PSA decline per month [14] or a decline in PSA at 12 weeks from the baseline [15] and prognosis. However, there is less evidence about the effect of early changes in PSA levels after beginning ADT in patients with mHSPC. PSA is easy and inexpensive to measure. In addition, PSA changes can be evaluated early with a clear cut-off level, unlike the nadir PSA.

In this study, we aimed to assess whether the early percent-change in the PSA level after 3 months of ADT treatment (%PSA) is a predictive marker for mHSPC patients treated with ADT.

## Main text

### Methods

#### Patients

We retrospectively reviewed the medical records of 71 consecutive primary mHSPC patients treated with ADT at the University of the Ryukyus Hospital or Naha City Hospital between January 2005 and June 2018.

#### Procedure

Local evaluations were performed by rectal examination, transrectal ultrasound, or magnetic resonance imaging (MRI). Evaluations of the regional lymph nodes were performed by computed tomography (CT) or MRI. Evaluations of distant metastasis were performed using CT, bone scintigraphy, or ^18^F-fluorodeoxyglucose-positron emission tomography/computed tomography (FDG-PET/CT).

Patients were considered to be on ADT if they were on any luteinizing hormone-releasing hormone (LHRH) agonists or LHRH antagonists, or had undergone surgical castration or combined androgen blockade (CAB). CAB included combinations of LHRH agonists or LHRH antagonists and flutamide or bicalutamide, or surgical castration and flutamide or bicalutamide. For drug treatments, we administered 11.25 mg leuprorelin acetate once every 3 months or 10.8 mg goserelin acetate once every 3 months as an LHRH agonist; we administered degarelix acetate initially at 240 mg and then once a month at 80 mg as an LHRH antagonist. We administered 125 mg flutamide three times daily or 80 mg bicalutamide once daily as an antiandrogen of CAB. CRPC was defined according to three criteria: (1) an increase in PSA levels based on the definition of prostate cancer working group 2 (PCWG2) [16], a ≥ 25% increase in PSA levels and an absolute increase of ≥ 2 ng/mL from the nadir, which was confirmed by a second value obtained ≥ 3 weeks later; (2) exacerbation on image evaluation; or (3) the judgment of the attending physician (including a change of drug).

The time to CRPC was defined as the period from the day ADT was started to the day CRPC was diagnosed. Overall survival (OS) was defined as the time from the start of ADT to the date of death from any cause. The factors evaluated for predicting the time to CRPC and OS included: the PSA value at diagnosis, Gleason score at biopsy, TNM classification before treatment, extent of disease (EOD) value, presence/absence of visceral metastasis, presence/absence of CAB therapy, presence/absence of bone modifying agents (BMA), and the 3-month %PSA. The patients were divided into two groups based on their median PSA level (≤ 261 ng/mL vs. > 261 ng/mL). The 3-month %PSA (median PSA: 1.1%) was used to divide patients into two groups: a group with ≥ 1% of the pretreatment levels (PSA ≥ 1%) and a group with lower PSA levels (PSA < 1%). An EOD ≥ 2 was defined by the presence ≥ 6 bone metastases [17].

### Statistical analysis

The statistical software used was JMP version 12, and the analyses of time to CRPC and OS were estimated using the Kaplan–Meier method and tested using the log rank test. Prognostic factors were analyzed by Cox proportional hazard regression. In univariate analysis, we included all clinical items. In multivariate analysis, we selected items that were considered clinically important according to previous reports [5, 6, 14, 15] or our univariate analysis, which included PSA levels ≥ 261 ng/mL, Gleason score ≥ 8, N stage, visceral metastasis, bone metastasis EOD ≥ 2, received BMA, and %PSA ≥ 1. A receiver operating characteristic (ROC) curve was utilized to assess the diagnostic accuracy of time to CRPC using three factors: a Gleason score ≥ 8, EOD ≥ 2, and a %PSA ≥ 1 according to multivariate analysis. All p-values < 0.05 were defined as statistically significant.

## Results

### Clinical characteristics of patients

The average patient age was 69.3 ± 8.1 years (mean ± SD), and the median PSA level at diagnosis was 261 ng/mL (interquartile range [IQR]: 92.5–618 ng/mL). A Gleason score ≥ 8 was found in 60 cases (85%). According to the TNM classification, 41 (58%) cases were classified as T3 or above, meaning more than half were locally progressive. Bone metastases were observed in 65 cases (92%), and visceral metastases were observed in 17 cases (24%). The median PSA level after 3 months of ADT was 2.6 ng/mL (IQR: 0.03–1092), and the median 3-month %PSA was 1.1% (Table 1).

**Table 1:** Patient characteristics

Age	69.3 ± 8.1
PSA (ng/mL)	261 (IQR: 92.5–618)
Gleason score	
8 or higher	60 (85%)
T stage	
1	1 (1%)
2	26 (37%)
3	24 (34%)
4	17 (24%)
Unknown	3 (4%)
N stage	
0	28 (39%)
1	43 (61%)
M stage	
Bone	65 (92%)
Visceral	17 (24%)
mCRPC	57 (80%)
Serum testosterone values (ng/dL)	12 (IQR: 6–21)
EOD score of 2 or more	38 (57%)
CAB therapy	69 (97%)
Used BMA	34 (48%)
Median PSA levels after 3 months of ADT	2.6 (0.03–1092)
Median PSA levels after 3 months of ADT (%)	1.1 (0.01–103.7)
Median observation periods (months)	38 (5–156)

### Background of CRPC progression and OS

The median observation period was 38 months (range: 5–156); during that period, 57 patients (80%) progressed to CRPC. The median times to CRPC and OS were 15 months (Additional file 1: Figure S1) and 92 months (Additional file 2: Figure S2), respectively.

In patients with EOD ≥ 2, the time to CRPC was significantly shorter (p = 0.033) than in patients with EOD ≤ 1. In the group of PSA ≥ 1% after 3 months of ADT, the time to CRPC was significantly shorter (p = 0.027) (Additional file 3: Figure S3); the OS was also significantly shorter (p = 0.01). Lymph node metastasis and visceral metastasis were not significant factors in either the time to CRPC or OS (Additional file 4: Table S1).

In multivariate analysis using Cox proportional hazards regression, a Gleason score ≥ 8 (p = 0.004), EOD ≥ 2 (p = 0.004), and %PSA ≥ 1 (p = 0.017) were found to be independent predictors of shortening the time to CRPC. In addition, %PSA ≥ 1 was an independent predictor (p = 0.004) for OS (Table 2).

**Table 2:** Multivariate analysis adjusted for potentially significant covariates in subset of N = 71

Covariates	Time to CRPC, HR (95%CI)	P-value	OS, HR (95%CI)	P-value
PSA levels ≥ 261 ng/mL	1.16 (0.61–2.22)	0.656	2.71 (0.55–13.26)	0.219
Gleason score, 8 or higher	3.55 (1.46–9.89)	0.004	7.58 (0.75–76.94)	0.087
N stage	1.58 (0.87–2.96)	0.138	3.93 (0.98–15.73)	0.053
Visceral metastasis	1.98 (0.93–4.03)	0.074	2.91 (0.51–16.71)	0.231
Bone metastasis, EOD 2 or more	2.49 (1.34–4.78)	0.004	0.68 (0.18–2.65)	0.583
Used BMA	1.04 (0.57–1.88)	0.900	1.17 (0.33–4.16)	0.802
PSA levels after 3 months of ADT, 1% or more	2.07 (1.14–3.82)	0.017	13.18 (2.34–74.38)	0.004

**Fig. 1:**
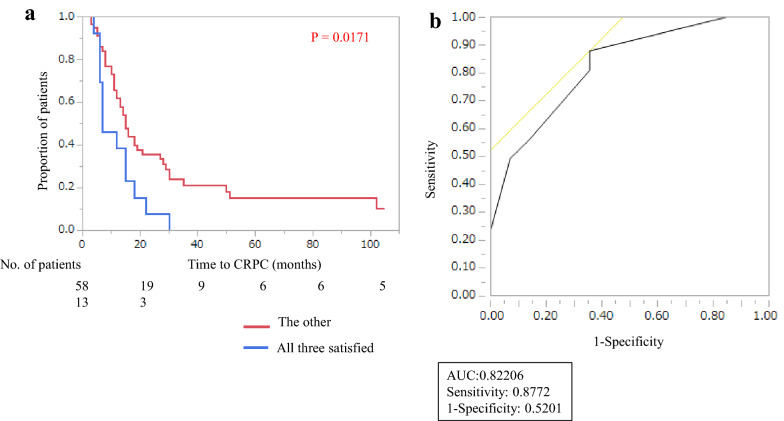
**a** Comparison of Kaplan–Meier curves between the high-risk group that satisfied three independent factors (Gleason score ≥ 8, EOD ≥ 2, and 3-month %PSA level ≥ 1) and the other group. The high-risk group shows a significantly shorter time to CRPC than the other group (p = 0.0171). **b** The AUC of CRPC diagnosis for differentiating the high-risk group and the other group is 0.822. That provides a sensitivity of 0.877 and a false-positive rate of 0.520

The time to CRPC was significantly shorter for the high-risk group, which satisfied three criteria (Gleason score ≥ 8, EOD ≥ 2, and 3-month %PSA ≥ 1) than for the other groups (p = 0.0171) (Fig. 1a). The area under the ROC curve (AUC) of CRPC diagnosis for differentiating the high-risk group and the other was 0.822. This provided a sensitivity of 0.877 and a false-positive rate of 0.520 (Fig. 1b).

## Discussion

In this study, we assessed the usefulness of early PSA change after ADT administration as a predictive marker for mHSPC patients. We found that %PSA ≥ 1 was a strong predictive factor for OS. In addition, Gleason score ≥ 8, EOD ≥ 2, and %PSA ≥ 1 were significantly associated with shorter time to CRPC. For diagnostic performance, the AUC of CRPC diagnosis for differentiating the high-risk group from the other group was feasible (0.822).

There have been several reports focusing on PSA-related variables, including initial PSA levels and PSA kinetics, which are the most frequently assessed biomarkers in mHSPC [18]. Among the PSA kinetic variables, nadir PSA and time to the nadir PSA are promising biomarkers for mHSPC [19, 20]. However, previous studies reported that the median time to the nadir PSA was 6–10 months [19, 20], which means that the prognosis was predictable only after more than half a year following the initiation of ADT. In contrast, in our report, the prognosis can be predicted at 3 months, and the judgment can be made in a short period. Ji et al. reported that a decline in PSA levels of > 11 ng/mL per month after initial ADT was significantly associated with an increase in the risk of progression to CRPC [14]. Therefore, to predict the outcomes of mHSPC patients, a 3-month %PSA might be a simple and convenient biomarker for the prediction of clinical outcomes. Sato et al. [15] also reported that a group whose PSA level decreased by 98.5% at 12 weeks after the initiation of ADT had significantly increased progression-free survival and OS. In our study, 3-month %PSA ≥ 1 was an independent predictive marker, consistent with Sato’s results. In their report, the proportion of patients with visceral metastases was relatively low at 3.3%. In contrast, in our report, 24% of patients had visceral metastases, which have relatively poor prognoses. It is interesting to note that, even in such patients, an early decline in PSA levels contributed to significantly longer OS. It is suggested that the 3-month PSA < 1% group may have a better prognosis in mHSPC.

In previous studies, serum bone markers [21], circulating tumor cells (CTC), [22] and single nucleotide polymorphisms [23] were identified as potential prognostic markers for patients with mHSPC. However, they are difficult and very expensive to adopt in clinical practice. In contrast, the early changes in PSA are simple and inexpensive to evaluate and are clinically useful.

The independent factors affecting the time to CRPC in our study were: (1) Gleason score ≥ 8, (2) EOD ≥ 2, and (3) %PSA ≥ 1. Two factors (Gleason score ≥ 8 and EOD ≥ 2), except for visceral metastasis, are compatible with the results of the initial trial of abiraterone acetate for high-risk mHSPC in Japan. In the LATITUDE trial [6], visceral metastasis was listed as an item of high risk; however, in our study, it was not an independent predictor of poor prognosis. Distant metastasis, particularly visceral metastasis, is an important negative prognostic factor in prostate cancers [3, 24–27]. Cui et al. reported that OS of prostate cancer patients with visceral metastasis with lung metastasis had a better prognosis than brain or liver metastasis [28]. In our study, 11 out of 17 patients (65%) with visceral metastasis had lung-only metastasis. This might be the reason that visceral metastasis was not shown to be a high-risk factor.

The present study reported that the group with 3-month %PSA ≥ 1 had poor prognoses, which suggests that it is a high-risk factor in mHSPC. In bone-metastatic prostate cancer patients, it is reported that age, T stage, PSA, Gleason score and EOD are useful as prognostic nomogram factors [29]. In our study, the AUC of the time to CRPC for differentiating the high-risk group that satisfied our three independent factors (Gleason score ≥ 8, EOD ≥ 2, and 3-month %PSA ≥ 1) was high at 0.822. Thus, our cut off level, 3-month %PSA ≥ 1, is feasible and might be useful for a new scoring method for detecting high-risk mHSPC.

## Conclusion

Three factors were independent predictors of shorter time to CRPC. Notably 3-month %PSA ≥ 1 correlates with a poor prognosis. These results suggest that the 3-month %PSA is a useful marker for predicting the prognosis of mHSPC.

## Limitations

Our study had several limitations. First, it is a retrospective analysis performed at two hospitals. As such, the number of cases is small and only from Asia, and there is a possibility that patient background and treatment selections might have been biased. Second, the judgment of the attending physician was used as one of the definitions of CRPC, and there is a possibility that the data was affected by the difference in judgments of the attending physicians.

## Supplementary Information

**Additional file 1: Figure S1:** Kaplan–Meier curve showing the time to CRPC (median: 15 months).

**Additional file 2: Figure S2:** Kaplan–Meier curve showing the overall survival (median: 92 months).

**Additional file 3: Figure S3:** Comparison of Kaplan–Meier curves between the PSA ≥ 1% group and the PSA < 1% group. The PSA ≥ 1% group shows a significantly shorter time to CRPC than the PSA < 1% group (p = 0.0027).

**Additional file 4: Table S1:** Univariate analysis of potential baseline covariates with the time to CRPC.

## Data Availability

The data supporting the conclusions used and/or analyzed in this study are available from the corresponding author by request.

## Abbreviations

PSA: Prostate-specific antigen
ADT: Androgen deprivation therapy
mHSPC: Metastatic hormone-sensitive prostate cancer
CRPC: Castration-resistant prostate cancer
OS: Overall survival
EOD: Extent of disease value
MRI: Magnetic resonance imaging
CT: Computed tomography
FDG-PET/CT: ^18^F-fluorodeoxyglucose-positron emission tomography/computed tomography
LHRH: Luteinizing hormone-releasing hormone
CAB: Combined androgen blockade
PCWG2: Prostate cancer working group 2
TNM: Tumor node metastasis
BMA: Bone modifying agents
