# Supplementary material for: Three-month early change in prostate-specific antigen levels as a predictive marker for overall survival during hormonal therapy for metastatic hormone-sensitive prostate cancer
Source: BMC Res Notes. 2021 Jun 3;14:227. doi: 10.1186/s13104-021-05641-5 (PMC8176613; doi:10.1186/s13104-021-05641-5)
Supplement: Supplementary file 1 — Additional file 1: Figure S1: Kaplan–Meier curve showing the time to CRPC (median: 15 months). [file 13104_2021_5641_MOESM1_ESM.pptx]

## Slide 1
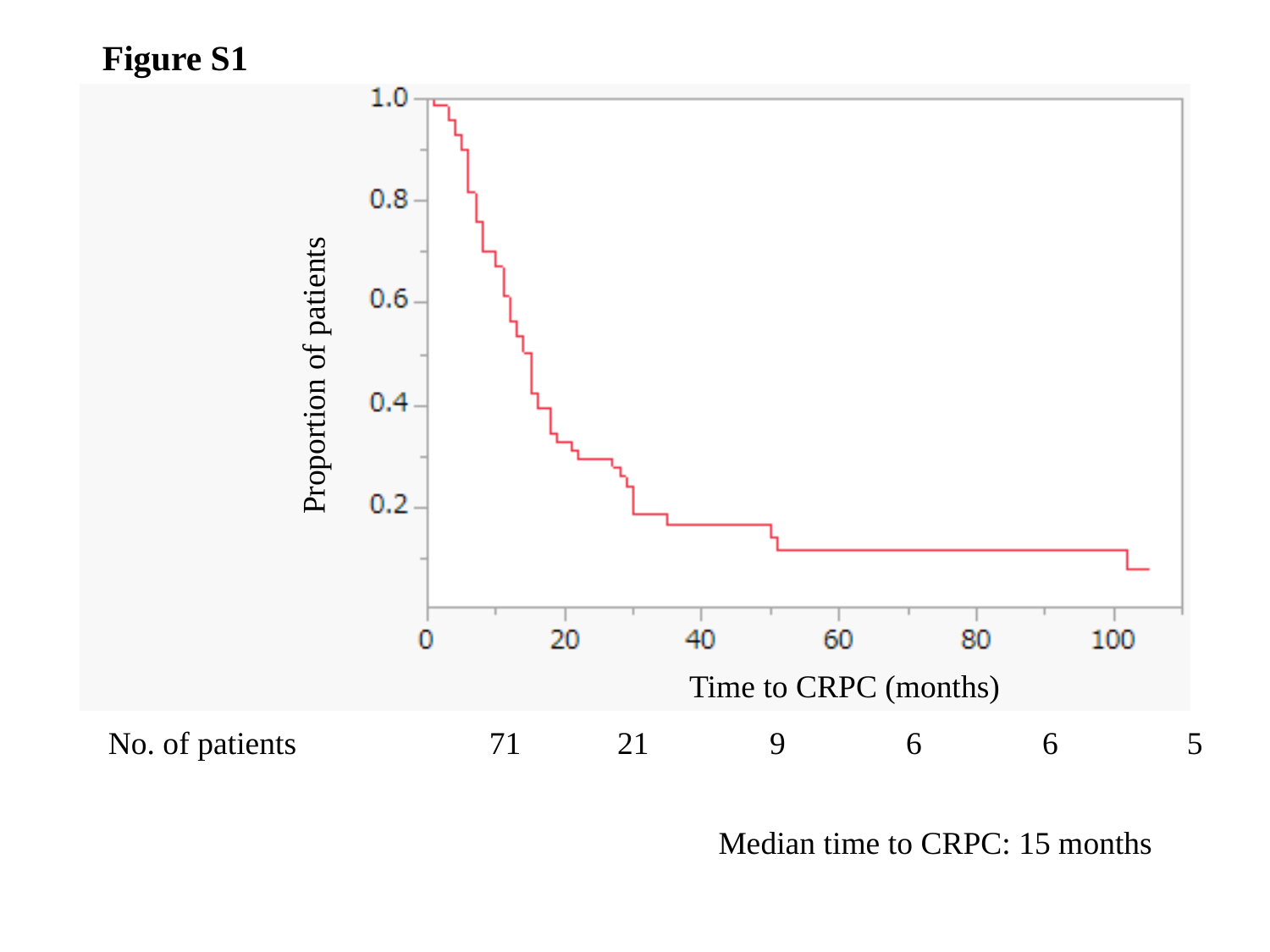

Figure S1
Proportion of patients
Time to CRPC (months)
No. of patients		71 21 9 6 6 5
Median time to CRPC: 15 months
