# Supplementary figures and images for: Three-month early change in prostate-specific antigen levels as a predictive marker for overall survival during hormonal therapy for metastatic hormone-sensitive prostate cancer
Source: BMC Res Notes. 2021 Jun 3;14:227. doi: 10.1186/s13104-021-05641-5 (PMC8176613; doi:10.1186/s13104-021-05641-5)

## Slide 1
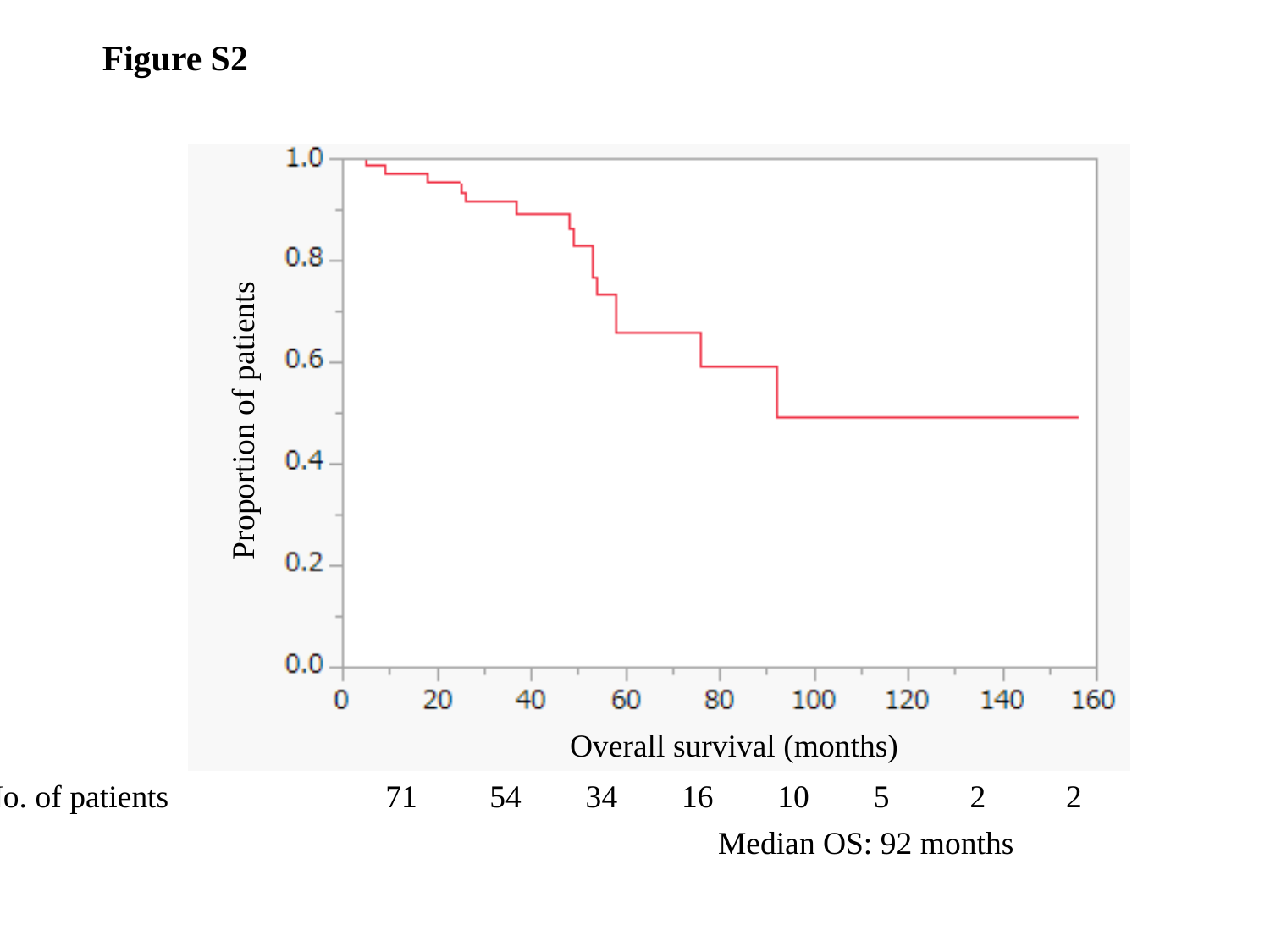

Figure S2
Proportion of patients
Overall survival (months)
No. of patients		 71 54 34 16 10 5 2 2
Median OS: 92 months

Supplement: Supplementary file 2 — Additional file 2: Figure S2: Kaplan–Meier curve showing the overall survival (median: 92 months). [file 13104_2021_5641_MOESM2_ESM.pptx]

## Slide 1
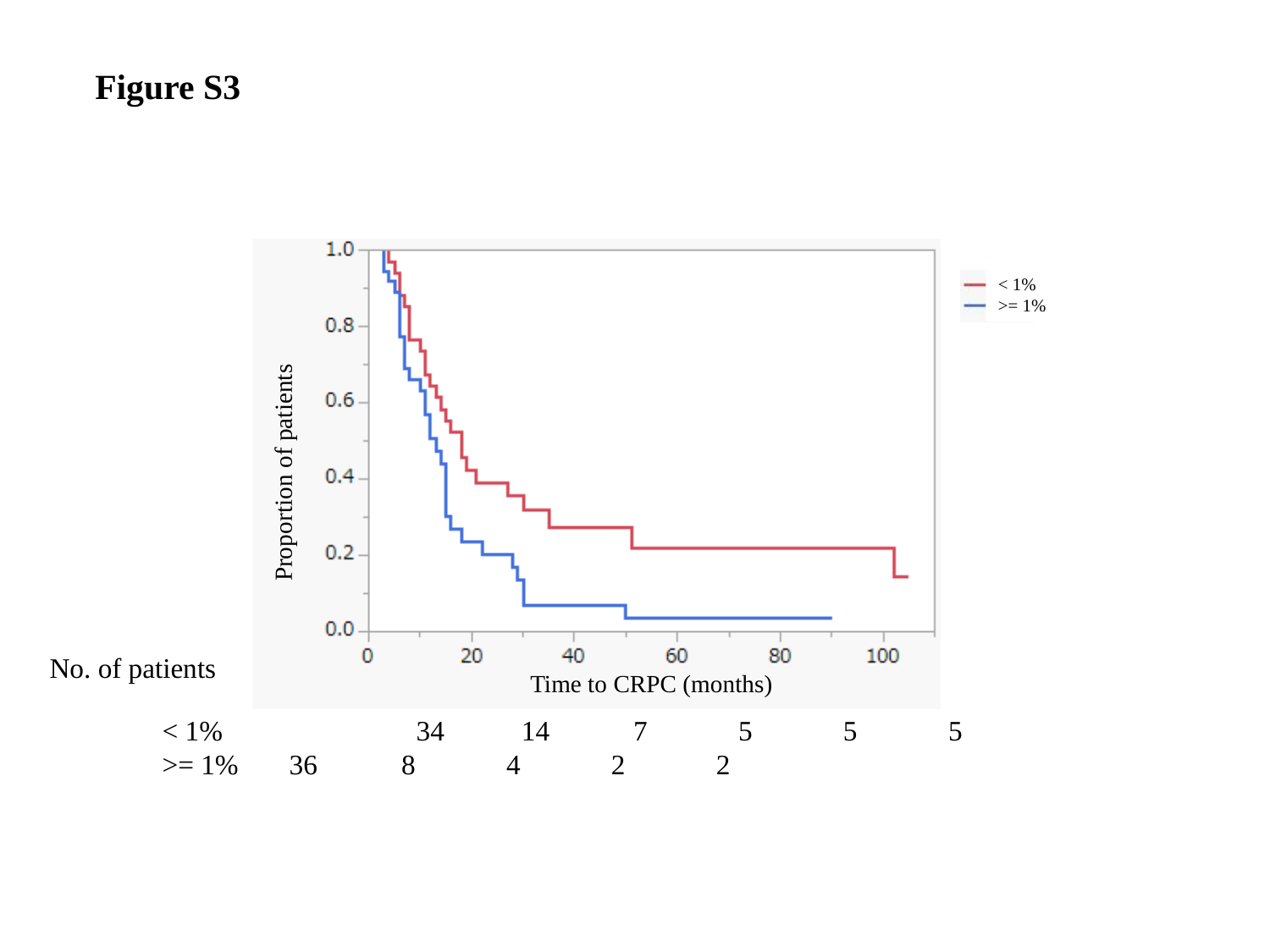

Figure S3
< 1%
>= 1%
Proportion of patients
No. of patients
Time to CRPC (months)
< 1%		34 14 7 5 5 5
>= 1%	36 8 4 2 2

Supplement: Supplementary file 3 — Additional file 3: Figure S3: Comparison of Kaplan–Meier curves between the PSA ≥ 1% group and the PSA < 1% group. The PSA ≥ 1% group shows a significantly shorter time to CRPC than the PSA < 1% group (p = 0.0027). [file 13104_2021_5641_MOESM3_ESM.pptx]
