# Supplementary material for: Three-month early change in prostate-specific antigen levels as a predictive marker for overall survival during hormonal therapy for metastatic hormone-sensitive prostate cancer
Source: BMC Res Notes. 2021 Jun 3;14:227. doi: 10.1186/s13104-021-05641-5 (PMC8176613; doi:10.1186/s13104-021-05641-5)
Supplement: Supplementary file 4 — Additional file 4: Table S1: Univariate analysis of potential baseline covariates with the time to CRPC. [file 13104_2021_5641_MOESM4_ESM.docx]

Supplementary Table S1

| Univariate analysis of potential baseline covariates with the time to CRPC and OS | | |  |  |
| --- | --- | --- | --- | --- |
|  |  |  |  |  |
| **Covariates** | **Time to CRPC, HR (95%CI)** | **P-value** | **OS, HR (95%CI)** | **P-value** |
| Age, 70 or more | 1.09 (0.65 – 1.83) | 0.753 | 1.98 (0.70 – 5.58) | 0.185 |
| PSA levels ≥ 261 ng/ml | 1.17 (0.69 - 1.98) | 0.553 | 1.08 (0.39 - 2.99) | 0.876 |
| Gleason score, 8 or higher | 1.74 (0.81 - 3.75) | 0.158 | 4.07 (0.53 - 31.14) | 0.177 |
| T stage, T3 or higher | 1.54 (0.88 – 2.69) | 0.133 | 1.67 (0.52 – 5.33) | 0.387 |
| N stage | 1.59 (0.91 - 2.75) | 0.101 | 2.38 (0.77 - 7.31) | 0.130 |
| visceral metastasis | 1.45 (0.80 - 2.63) | 0.218 | 1.65 (0.46 - 5.92) | 0.446 |
| bone metastasis, EOD 2 or more | 1.80 (1.05 - 3.10) | 0.033 | 0.94 (0.34 - 2.62) | 0.911 |
| Used BMA | 0.79 (0.47 - 1.34) | 0.389 | 0.51 (0.18 - 1.44) | 0.205 |
| PSA levels after 3 months of ADT, 1% or more | 1.84 (1.07 - 3.16) | 0.027 | 7.05 (1.58 - 31.41) | 0.010 |
